# Chromogenic Black Dental Staining in Children: A Case Report

**DOI:** 10.7759/cureus.51984

**Published:** 2024-01-09

**Authors:** Sattam T Alammari, Fahad M Al Rubaie, Bandar S Shukr

**Affiliations:** 1 Dentistry, Taif University, Taif, SAU

**Keywords:** pediatric dentistry, extrinsic discoloration, chromogenic bacteria, chromogenic staining, black stains

## Abstract

*Chromogenic black dental staining* is an extrinsic dental discoloration seen in daily dental practice, especially in children. These stains are commonly seen as black dots or lines along the cervical third of the teeth. They can affect both primary and permanent dentitions. The evidence is contradictory regarding its etiology, with links to different microbial, dietary, and iatrogenic factors. It was suggested that the back discoloration contains traces of insoluble ferric salt due to the interaction between hydrogen sulfide-producing chromogenic bacteria and iron in the saliva or gingival secretions. The current article aims to overview black dental staining in children and present a case report on a pediatric patient.

## Introduction

Dental dyschromia, often known as tooth discoloration, is a major dental condition that can cause aesthetic problems in individuals of all ages [1,2]. It could substantially affect a person's self-confidence and personality, especially in children [3]. Several systemic and local etiological factors might contribute to tooth discoloration [4]. In addition, tooth discoloration can be classified into intrinsic and extrinsic discolorations [1]. Intrinsic tooth discoloration is a severe form of dental staining that occurs due to changes to the internal structural composition or thickness of the tooth's hard tissues (enamel or dentine or both) [2]. Common etiological factors for this type of discoloration include the use of certain substances or medications (e.g., tetracycline, excessive fluoride intake) and genetic disorders (e.g., amelogenesis imperfecta, dentinogenesis imperfecta) [2].

Extrinsic dental discoloration results from the accumulation of chromogens, which are colorless substances that can change color chemically on the outer layer of enamel or the salivary pellicle that covers teeth [5]. One of the most common forms of extrinsic discoloration is black stain.

Exogenous tooth discoloration, chromogenic, or pediatric staining are other terms used to refer to black stains [2]. Black stains are usually characterized by black dots or lines along the gingival third of both primary or permanent teeth, which are hard to remove using a toothbrush and may reappear after clearing [1]. The current evidence regarding the prevalence of black stains is mixed, with no evident sex predilection [2,6,7]. The prevalence has been reported to be between 2 to 20% [2], with the highest prevalence (around 20%) found in 7-15 years old children from Basel, Switzerland [8], and the lowest (2.5%) was among 3-5 years old children from Brazil [9]. The variability of the prevalence in different investigations could be attributed to the different lifestyles, environments, and habits among the different study populations, which might be one of the potential etiological factors for chromogenic staining [10].

It is unclear what causes black stains; however, they were linked to different microbial, dietary, and iatrogenic factors [1,2]. Microscopically, black stains possess a unique form of dental plaque or biofilm highly susceptible to calcification [6]. Its microbial composition is believed to contain chromogenic bacteria, including actinomyces and prevotella melaninogenic [11]. It has been suggested that the back discoloration contains traces of insoluble ferric salt due to the interaction between hydrogen sulfide-producing bacteria and iron in the saliva or gingival fluid [3,6,11]. Therefore, the literature suggests an increased likelihood of developing black stains in people who frequently consume a diet rich in iron and in individuals who use iron supplements, particularly during early childhood and pregnancy [12]. Additionally, consuming vegetables, fruits, dairy products, eggs, and soy sauce encourages the development of black stains [7].

Clinically, black stains are identified as non-cavitated extrinsic black discolorations that commonly affect the teeth' buccal and palatal/lingual areas [6]. They appear as dark, pigmented lines that run parallel to the gingival margin or as an incomplete connection of dark dots [12]. These stains affect both deciduous and permanent teeth and are usually located at the cervical part, with rare extension to the proximal surfaces [2,6]. Occasionally, these stains may also affect the dental pits and the base of the grooves [1]. Research indicates that the lingual surfaces of lower anterior teeth are the most affected areas [7]. This is probably because they are in an environment that is rich with saliva (near the opening of mandibular salivary glands), as saliva was shown to be one of the possible etiological factors for black stains [7]. During clinical examination, it's critical for the examiner to distinguish between black stains and dental caries [2]. Since black staining is an accumulation on the tooth's undamaged surface, it can be eliminated with prophylactic cleaning or polishing [2]. On the other hand, dental caries is an irreversible destruction of the enamel, dentine, or both, which requires restorative treatment [2]. Moreover, the distinctive dotted line, which is linearly confined to the gingival border, helps distinguish black staining from dental caries.

Currently, there are three different systems to classify black stains. Shourie developed the first in 1947 [13], which included three scores: score (1) means no pigmentations; score (2) means incomplete coalescence of pigmented spots; and score (3) indicates a continuous line of pigmented spots. The system was later modified by Koch et al. in 2001 by including an additional diagnostic criterion: the presence of pigmented linear spots parallel to the gingival margin in at least two different teeth without enamel cavitation [8]. In 2003, the classification was further modified by Gasparetto et al. to include new criteria based on the extension of the surface area in the affected tooth [14]. Gasparetto's classification is considered the most recent one, and it classified black stains into three different scores: score 1 (pigmented spots or thin lines parallel to the gingival border that are incompletely coalescing); score 2 (continuous pigmented lines that only cover half of the tooth's cervical third); and score 3 (pigmentations that cover more than half of the tooth's cervical third).

## Case presentation

**Figure 1:**
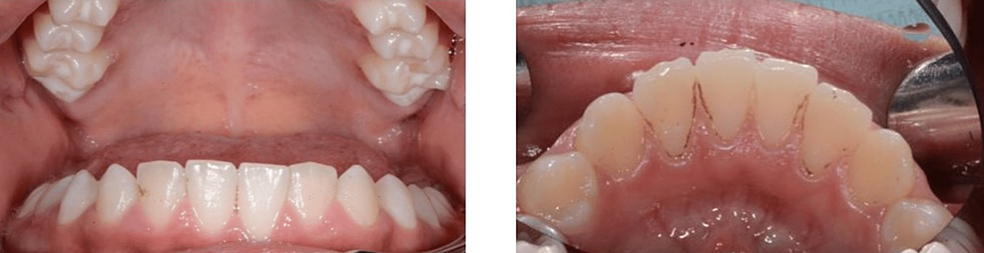
Labial and lingual views of the lower anterior teeth before treatment showing the black stains in the labial, lingual, and proximal surfaces.

Accompanied by his parents, an 11-year-old male child attended the Speciality Paediatric Clinics at the University Dental Hospital (UDH) in Taif University with a complaint of pain in the upper right quadrant. No issues were detected when past medical and family histories were reviewed. Dental history showed a good oral hygiene regime with twice-a-day fluoridated toothpaste brushing under parental supervision. Extra-oral examination was within normal range. The dietary analysis revealed high consumption of unhealthy diets. Upon intra-oral examination, the examiner noticed a black staining affecting the lower anterior teeth in the labial, lingual, and proximal surfaces (Figure 1). Most of these stains were classified as Gasparetto's score (3), as they were extended beyond half of the cervical part of the teeth.

**Figure 2:**
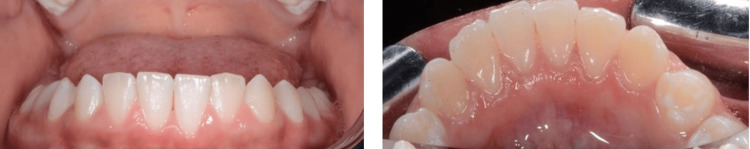
Labial and lingual views of the lower anterior teeth after treatment showing successful removal of the black stains.

The parents were reassured about the nature of the chromogenic staining and its expected short-term occurrence as part of the treatment plan, which was approved by signing the informed consent. Furthermore, the importance of consuming a healthy balanced diet was highlighted, in addition to explaining the other potential causes of chromogenic staining. The treatment involved professional teeth cleaning and polishing using an ultrasonic scaler, prophy pastes, and rubbers. The treatment session lasted approximately 45 minutes, and all the pigmentations were successfully removed from the teeth (Figure 2). The child and the parents were reminded about the possible recurrence of these stains, and a recall appointment was scheduled before they were dismissed. A follow-up examination three months after the initial treatment revealed no recurrence of these stains. Black stains may quickly return after removal, especially if the patient ignores the dietary and oral hygiene recommendations. Therefore, these recommendations were emphasized again during the recall visit.

## Discussion

Chromogenic staining may be encountered in daily practice, with greater prevalence found among children [15]. Until now, the evidence regarding its etiology and epidemiology is mixed, with some research showing a higher risk of staining among children who have never been fed with a nursing bottle [1,2]. The pigmentations can disappear if developed on primary teeth, as the teeth will eventually be replaced by their permanent counterparts. Nevertheless, the first step in the comprehensive treatment of chromogenic staining is to inform the child and the parents or legal guardian about the potential causes of these satins and reassure them that they are not permanently developed on the teeth [2]. Additionally, they should be instructed to avoid aggressive brushing habits when brushing to prevent cavity formations due to abrasion [2]. The typical treatment involves the removal of these satins in a professional session using a combination of scaling and polishing procedures to satisfy the child's aesthetic needs, in addition to dietary consultations [2]. It is critical for the treating dentist or hygienist to avoid the overuse of ultrasonic scalers because it may result in an unwanted loss of the enamel layer [2].

The professional cleaning can be repeated if the pigmentations reappear in the follow-up visit [2]. In severe cases, other treatment options could be considered, such as micro-abrasion with abrasives (i.e., air-jet polishing) [16], photodynamic therapy (e.g., diode laser, laser nd: YAG) [17], antimicrobial therapy (i.e., Lactoferrin and lactoperoxidase tablets) [18], and the use of bleaching agents [19]. Furthermore, a new emerging therapy to prevent the development or recurrence of chromogenic staining is using an oral probiotic, particularly Streptococcus salivarius M18 (SsM18) [20]. This type of therapy works by achieving a balanced state of microbiota, therefore improving oral health [20]. The findings revealed a successful reduction in the recurrence of staining in a clinical trial conducted in children [20].

Successful treatment of black staining is still a challenge for dentists and dental hygienists because black staining has a high chance of recurrence after professional treatment. Hence, it is critical to educate both the child and the parents about the importance of practicing good oral hygiene habits and controlling the other causative factors, such as iron-rich foods and supplements.

## Conclusions

Black chromogenic stains are extrinsic dental discolorations in daily dental practice, especially in pediatric patients. They can disturb the aesthetic appearance of the child and affect his/her personality and self-esteem. It is crucial for both dentists and dental hygienists to develop good knowledge about black stains to ensure accurate diagnosis and appropriate treatment or referral to a specialized pediatric clinic. When removing these stains, the clinician should pay attention to not causing iatrogenic harm to the tooth or other oral tissues.
